# Biologics for Non-Cancer Dermatological Diseases: Analysis on China’s Clinical Trial Registration Trend From 2016 to 2020

**DOI:** 10.3389/fphar.2022.817065

**Published:** 2022-04-21

**Authors:** Beibei Zhu, Yi Liu, Jing Li, Qi Ni, Zheng Yin, Junli Zhu, Ken Chen, Xueyuan Yang

**Affiliations:** ^1^ Institute of Dermatology, Chinese Academy of Medical Sciences and Peking Union Medical College, Nanjing, China; ^2^ Real World Solutions, IQVIA, Shanghai, China

**Keywords:** dermatology, biologics, China, clinical trial, trend

## Abstract

**Background:** In the past 5 years, China has quickly followed US’s steps to approve the new drug application of biologics for dermatological diseases. There is an increasing interest in the current biologic landscape and further potentials in China. Our study aims to analyze features of clinical trials on non-cancer dermatological biologics and synthesize recent achievements and impediments, in order to forecast the development trends in China.

**Methods:** Three registers (the Chinese Clinical Trial Registry, Center for Drug Evaluation, and ClinicalTrials.gov) were searched for clinical trials of non-cancer dermatological biologics initiated between 2016 and 2020 in mainland China. Trial information (the first posted year, sponsor type, study phase and site, recruitment status, disease, drug target, and drug registration type) and certain study design (selection of the control group, primary efficacy outcome, and inclusion of patient-reported outcome for non–phase I or II trials only) information were extracted and analyzed.

**Results:** A total of 60 trials were identified. The number of registered dermatological biologic trials significantly increased with an annual increase of 59% from 2016 to 2020, primarily driven by psoriasis (47/60, 78.3%) and atopic dermatitis (6/60, 10.0%) trials. The tumor necrosis factor (TNF)-α and interleukin (IL)-17 remained the hottest drug targets (17/60, 28.3% for TNF-α and 18/60, 30.0% for IL-17). In addition to TNF-α and IL-17, many new psoriasis drug targets came into place since 2018 (IL-12/23) and 2019 (IL-36 and glucagon-like peptide-1 (GLP-1)). Thirty percent (18/60) of the trials were conducted for biosimilar products, all of which were sponsored by local pharmaceutical companies and 88.9% of which were targeting on TNF-α. Targets of IL-36, IL-5, and IgE were only available in trials sponsored by global companies.

**Conclusion:** There was great progress on the innovation of dermatological biologics in the past 5 years in China in terms of surged number of clinical trials, increased biosimilars and “me-too” drugs which greatly improved patient access to novel treatments, execution of parallel clinical trials, and improved hospital GCP office and regulatory environment. Further efforts for local pharmaceutical companies should include relocating resources to exploring novel drug targets and dermatological diseases other than psoriasis or atopic dermatitis.

**Systematic Review Registration**: [website], identifier [registration number].

## Introduction

Dermatological disease yields a high disease burden globally. Global burden of disease (GBD) showed that as of 2019, dermatological diseases ranked fourth place among burden of non-fatal diseases globally, affecting a third of the population (Institute for Health Metrics and Evaluation, 2019). In China, the prevalence of dermatological diseases was 40–70%, according to the “Expert Consensus on the Methodology of Dermato–Epidemiological Investigations” (Chinese Medical Association, 2020).

The visible nature of dermatological diseases, symptoms, and sequelae all contribute physically and psychosocially to the overall burden of disease, including negative impacts on the patient quality of life, social life, and productivity (Jowett and Ryan, 1985; Wu and Cohen, 2019). The direct and indirect costs of disease management also pose a heavy burden on the patient’s family and the society. For example, an atopic dermatitis patient survey conducted in 2020 showed that 22% of patients spent over CNY 12,000 in atopic dermatitis treatment in the past year, and 68.7% of patients had one to three outpatient visits per month. Of the patients with severe atopic dermatitis, 46.5% found it hard to work or study normally, 45.8% had challenges in performing social activities, and over 10% had suicidal intentions (China Association of Health Promotion and Education, 2020). Another psoriasis patient survey conducted in 2018 showed that the total annual expenditure for psoriasis per patient accounted for 20% of the total annual income, the annual hospitalization rate was 21.3%, the annual sick leave or absence duration was 15.0 days, and the unemployment rate due to psoriasis was 37.0% (Chen, et al., 2019). Along with population aging and emerging novel treatment, the economic burden of dermatological diseases is expected to increase over time.

Biologics include a wide range of products such as vaccines, blood and blood components, allergenics, somatic cells, gene therapy, tissues, and recombinant therapeutic proteins (US Food and Drug Administration, 2018). The target-directed feature of biologics renders unique efficacy and safety profile compared with traditional small-molecule drugs. As the first dermatological biologic, etanercept was approved for psoriasis by the Food and Drug Administration (FDA) in 1998. Since then, biologics have been increasingly playing an important role in the management of multiple dermatological diseases. In the past 5 years, a total of eight innovative drugs have been approved in China for non-cancer dermatological diseases, six of which were biologics, with an average gap of 2 years following their initial approvals for the same indication by the FDA. The rapid approval of innovative dermatological biologics reflected the improved accessibility to the state-of-the-art treatment in China. There is still a big gap between the large and increasing number of patients with dermatologic diseases in China and low access to biologics.

In this situation, a comprehensive review of industry updates and current trends on dermatological biologics is not only essential for pharmaceutical companies to support cost-effective research and development (R&D) but also for drug regulatory authorities to achieve smart governance.

As part of one of a series of reviews on drugs R&D in China by a multidisciplinary team, this study aims to analyze features of clinical trials on non-cancer dermatological biologics, assess novel agents currently being investigated, and forecast the development trends in China. The findings will highlight the potential for a precision medicine approach to enable prevention and more effective long-term control of this complex therapeutic area.

## Methods

### Search Strategy and Selection Criteria

We searched the Chinese Clinical Trial Registry (ChiCTR), Center for Drug Evaluation (CDE), and ClinicalTrials.gov on 22 September 2021 for clinical trials of non-cancer dermatological biologics initiated between 2016 and 2020 in mainland China. For CDE and ChiCTR, only indication (dermatological diseases) and first posted date (1 January 2016 to 31 December 2020) were applied. For ClinicalTrials.gov, the country (China) and study type (interventional study) were applied in addition to indication and first posted date.

Selection of clinical trials was performed by two independent researchers. The inclusion criteria were the following: 1) focusing on non-cancer dermatological diseases defined by ICD-11 (World Health Organization, 2021), 2) biologics as intervention, 3) interventional studies as the study type, and 4) at least one study site in mainland China. The exclusion criteria were the following: 1) vaccines or cell therapy as intervention, 2) non-systemic therapy as intervention, and 3) duplicate registration in more than one register.

### Data Extraction

Data extraction was performed by two independent researchers. The following data were extracted: 1) the first posted year, sponsor type (global or local pharmaceutical company or investigator), study phase (I, II, III, IV, or others), study site (global or local), and recruitment status (active, completed, suspended/withdrawn, or unknown); 2) disease, drug target, and drug registration type by the National Medical Products Administration (NMPA) (innovative, modified, or marketed biologics for phase I to III trials only) (National Medical Products Administration, 2020). Since phase I or II trials have little flexibility on the control group and outcome selection, the following data were extracted for non–phase I or II trials only: 1) control group (placebo/no treatment or active control), 2) primary efficacy outcome, and 3) patient-reported outcome (PRO).

### Data Analysis

Descriptive analysis was performed on all collected variables by year from eligible trials. Drug targets were further summarized by disease, drug registration type, and trial phase to forecast biologic marketing. The sponsor type by the drug registration type and drug target was also descriptively summarized.

## Results

### Time Trend and Features of Trials on Biologics for Dermatological Diseases

**FIGURE 1:**
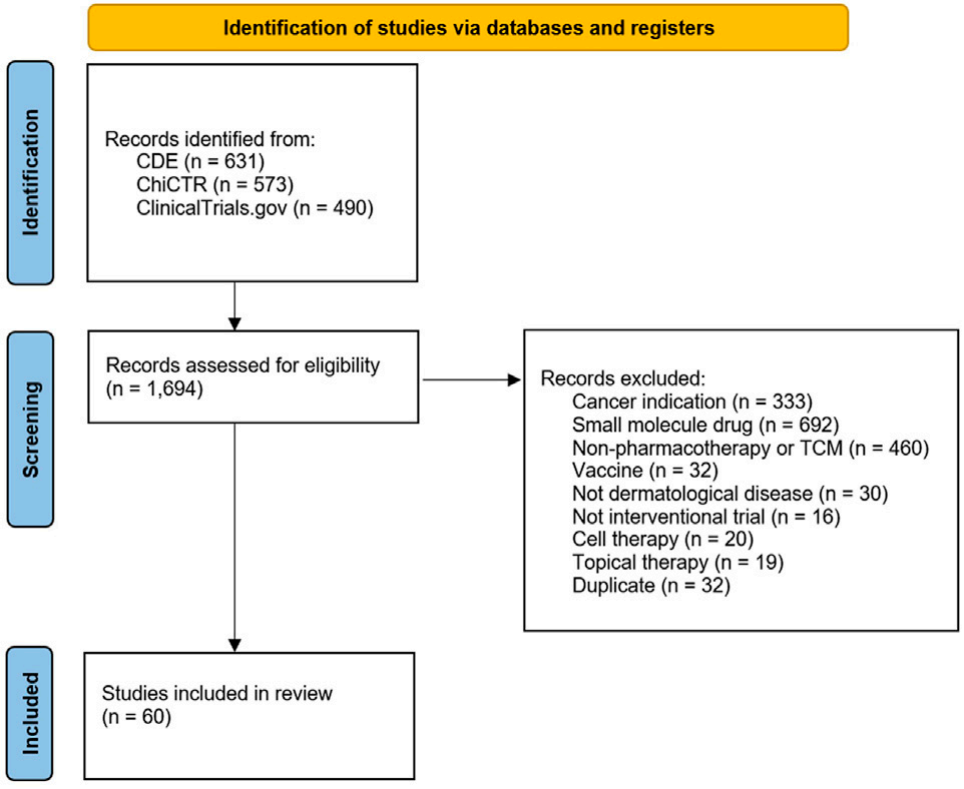
PRISMA flow diagram.

**FIGURE 2:**
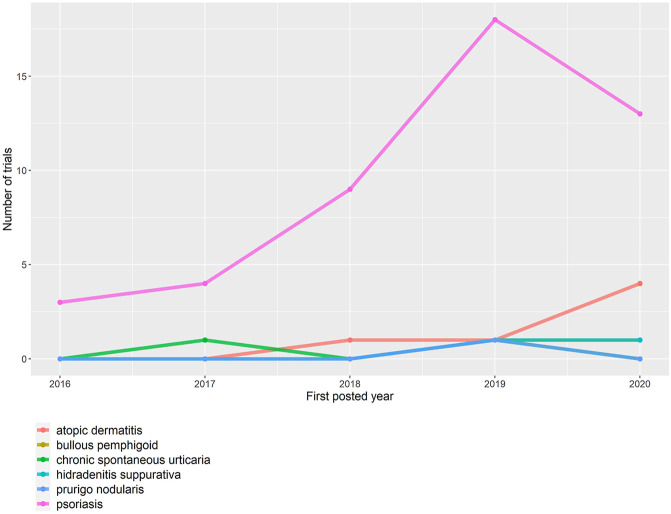
Time trend of dermatological biologic trials in China by disease type.

A total of 1,694 records (490 from ClinicalTrials.gov and 1,204 from CDE and ChiCTR) between 2016 and 2020 were initially retrieved, regardless of indication and intervention (Figure 1). Of these, 60 trials were conducted in China to investigate biologics for dermatological diseases. The majority of trials were sponsored by local (43/60, 71.7%) pharmaceutical companies, conducted solely in mainland China (51/60, 85.0%) and conducted for brand products (42/60, 70.0%). These trials mainly focused on biologics for psoriasis (47/60, 78.3%), followed by atopic dermatitis (6/60, 10.0%), bullous pemphigoid (2/60, 3.3%), chronic spontaneous urticaria (CSU) (2/60, 3.3%), hidradenitis suppurativa (2/60, 3.3%), and prurigo nodularis (1/60, 1.7%) (Figure 2). Over half of the trials (51.7%, 31/60) were phase I, 10 (16.7%) were phase II, 15 (25.0%) were phase III, and 4 (6.7%) were phase IV or others. One phase II and one phase IV trial were suspended or withdrawn, respectively, because of the following reasons: 1) sponsor’s decision and 2) reconsideration of business in China (Table 1).

**TABLE 1:** Characteristics of dermatological biologic trials by year (2016–2020).

	2016	2017	2018	2019	2020	All (%)
Sponsor type
Global company	0	3	2	5	4	14 (23.3)
Local company	3	2	8	15	15	43 (71.7)
Investigator	0	0	0	3	0	3 (5.0)
Study phase						
Phase I	2	1	6	12	10	31 (51.7)
Phase II	0	1	1	4	4	10 (16.7)
Phase III	1	3	3	4	4	15 (25.0)
Phase IV and others	0	0	0	3	1	4 (6.7)
Study site						
Global	0	2	0	4	3	9 (15.0)
Local	3	3	10	19	16	51 (85.0)
Recruitment status						
Completed	0	3	7	7	7	24 (40.0)
Active	1	0	2	15	11	29 (48.3)
Suspended/withdrawn	0	0	0	1	1	2 (3.3)
Unknown	2	2	1	0	0	5 (8.3)
Drug type						
Brand	3	4	5	16	14	42 (70.0)
Biosimilar	0	1	5	7	5	18 (30.0)
Disease						
Psoriasis	3	4	9	18	13	47 (78.3)
Atopic dermatitis	0	0	1	1	4	6 (10.0)
Bullous pemphigoid	0	0	0	1	1	2 (3.3)
Hidradenitis suppurativa	0	0	0	1	1	2 (3.3)
Prurigo nodularis	0	0	0	1	0	1 (1.7)
Chronic spontaneous urticaria	0	1	0	1	0	2 (3.3)
Drug target						
TNF-α	1	1	5	6	4	17 (28.3)
IL-17	1	3	3	6	5	18 (30.0)
IL-12/23	0	0	1	2	1	4 (6.7)
IL-23	0	0	0	1	1	2 (3.3)
IL-36	0	0	0	2	2	4 (6.7)
IL-4/13	0	0	1	2	4	7 (11.7)
IL-2	0	0	0	1	0	1 (1.7)
IL-5	0	0	0	0	1	1 (1.7)
CD6	1	0	0	0	0	1 (1.7)
C5a	0	0	0	1	1	2 (3.3)
GLP-1	0	0	0	1	0	1 (1.7)
Non-specific	0	0	0	1	0	1 (1.7)
Drug registration type (phase I to III only)						
Type I (innovative biologic)	3	1	4	12	11	31 (55.4)
Type II (modified biologic)	0	0	0	1	1	2 (3.6)
Type III (marketed biologic)	0	4	6	7	6	23 (41.1)

The number of registered dermatological biologic trials significantly increased over time until 2019 and slightly decreased in 2020 (3 in 2016, 5 in 2017, 10 in 2018, 23 in 2019, and 19 in 2020) (Table 1). The trend of dermatological biologic trials was primarily driven by psoriasis trials (3 in 2016, 4 in 2017, 9 in 2018, 18 in 2019, and 13 in 2020).

### Target by Disease and Trial Phase

**FIGURE 3:**
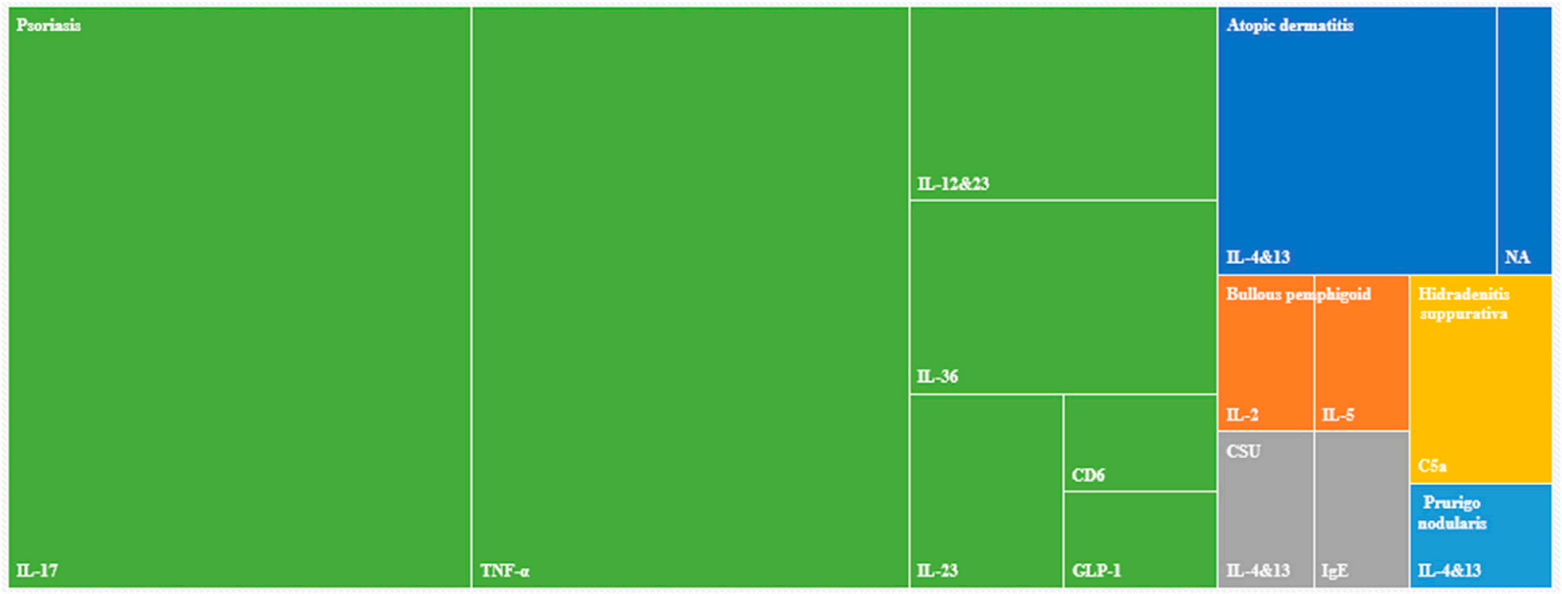
Distribution of biologic target and disease.

The tumor necrosis factor (TNF)-α and interleukin (IL)-17 remained the most common drug targets (17/60, 28.3% for TNF-α and 18/60, 30.0% for IL-17) in the past 5 years, and all trials on TNF-α and IL-17 were for psoriasis. Since 2018, trials in China started to investigate IL-12/23 (all four trials for psoriasis) and IL-4/13 (5/7 for atopic dermatitis, one for prurigo nodularis and one for CSU). The variety of targets increased in 2019 and 2020, including IL-36 (all four trials for psoriasis), IL-23 alone (two for psoriasis), IL-2 (1, bullous pemphigoid), IL-5 (1, bullous pemphigoid), GLP-1(1, psoriasis), complement component 5a (C5a, 1, hidradenitis suppurativa), and immunoglobulin E (IgE, 1, CSU). Only one phase I trial in 2016 investigated cluster of differentiation 6 (CD6) (Figure 3).

For psoriasis targets, most phase III trials studied TNF-α (n = 7), IL-17 (n = 2), and IL-23 (n = 1). Other targets in phase III trials included IL-4/13 (n = 1 for atopic dermatitis, prurigo nodularis, and CSU, respectively), IL-5 (n = 1 for bullous pemphigoid), and IgE (n = 1 for CSU). In terms of phase I trials, TNF-α and IL-17 accounted for 32.3 and 38.7% of the 31 phase I trials, respectively. C5a and CD6 were only studied in phase I trials.

### Sponsor Type by the Drug Registration Type and Drug Target

Of the 13 phase I to III trials sponsored by global companies, four (30.8%) were for type 1 (innovative biologics) registration, while the percentage of type 1 registration for local companies was 55.8% (24/43). Of the innovative biologic trials on IL-17, 93.3% (14/15) were conducted by seven local companies. Of the innovative biologic trials on IL-4/13, four out of six trials were conducted by three local companies. All four trials on IL-36 for psoriasis were conducted by the original global pharmaceutical company. In contrast, the vast majority (16/17) of trials on TNF-α were biosimilars, and half (7/16) were biosimilars of adalimumab by six local companies.

### Study Design of Non–Phase I or II Trials

In terms of study design for 19 non–phase I or II trials, only one trial did not have a control group. Six trials utilized an active treatment control, while the other 12 trials were controlled by placebo or no treatment. Selection of primary efficacy outcomes was dependent on the disease. For psoriasis, all trials utilized the Psoriasis Area and Severity Index (PASI) 75 or percentage improvement of PASI score from the baseline as their primary efficacy outcomes. Two trials used the Static Physician Global Assessment (sPGA) Score or the Investigator’s Global Assessment (IGA) Mod 2011 scale as their primary efficacy outcomes in addition to the PASI. Fourteen (73.7%) trials chose PRO as their outcomes, mostly the Dermatology Life Quality Index (DLQI).

## Discussion

As a national-level pioneering exploration of the overview of clinical trials through three clinical trial registers that are most commonly used by Chinese researchers, this review provides comprehensive evidence for policy makers on the R&D of dermatological biologics in China and provides methodological reference to international peers. Our findings can effectively inform the smart R&D of pharmaceutical companies, reduce unnecessary investment, and optimize the allocation of medical resources in the healthcare system.

Our study reported a significant increase in the number of registered dermatological biologic trials over the past 5 years in China, with a slight drop in 2020. Over three-fourth (47/60) of the trials on biologics focused on psoriasis. Five key hotspots were identified. First of all, two in every five trials on biologics for psoriasis focused on IL-17 (18/47), with four global companies and seven local companies competing in this field in China. Second, four early phase trials on IL-12/IL-23 for psoriasis (4/47) were registered by three local companies. Third, a global company registered four phase I and phase II study trials in China on a first-in-class treatment approach, IL-36, for the treatment of flares in generalized pustular psoriasis (GPP), a rare form of psoriasis. Early involvement of China in the R&D indicates that the China market plays an important role in the development strategy for this biological therapy. Fourth, pipelines on biosimilars of TNF-α were another hotspot in the drug for psoriasis accounting for 36% (17/47) due to the patent expiration of adalimumab. Finally, IL-4 and 13 accounted for 11.7% of trials, especially for atopic dermatitis. Other biological targets were sparse with one or two trials in the recent 2 years.

The increasing trend of biologics for dermatological treatment in China reflects both the unmet needs of a large dermatological patient population, effects of the recent series of pharmaceutical sector reforms as a result of the 13th Five-Year Plan (FYP) of China (National Development and Reform Commission, 2015) and the global trend of biologic development for dermatological diseases. Considering psoriasis as an example, only 6.18% Chinese psoriasis patients were receiving biologics as per a survey in 2019 (Huang and Chen, 2021), compared to 17.7% in the United States (Lebwohl, et al., 2016). This gap drew great attention from both global and local pharmaceutical companies. Meanwhile, the 13th FYP guided that biologics, including monoclonal antibodies (mAb), protein- and peptide-based biotech drugs, therapeutic vaccines, and biotherapeutics, should be prioritized for accelerated access (National Development and Reform Commission, 2015). The Chinese-produced Yisaipu of Sunshine Guojian has the first-mover advantage and has been included in the National Reimbursement Drug List (NRDL) of China in 2017. Both Humira^®^ (adalimumab, TNF-α) and Cosentyx^®^ (secukinumab, iL-17) have been included in the NRDL in 2019 and 2020, respectively, with an over 50% drop in the price and a sharp increase in the sales in the following years (National Healthcare Security Administration, 2019; National Healthcare Security Administration, 2020). The inclusion of dermatological biologics by NRDL can significantly reduce economic burden on patients, improve the access to novel treatment options of Chinese patients, and consequently motivate substantial drug development. New “default system” on the procedure of Clinical Trial Application (CTA) by NMPA in 2018 allowed a drug clinical trial if a negative or questionable comment from the CDE has not been received within 60 days from the application (National Medical Products Administration, 2018). As a result, the average start-up period of drug clinical trials was significantly decreased from 12–16 months (Pharmaphorum, 2016) to almost 2 months. This may account for the sharpest increase of number of trials occurring between 2017 and 2019, when the number of trials went up by 4.5-fold. The slight drop in 2020 may suggest a limited negative impact until now from the coronavirus disease–19 pandemic to the drug development in China, which is consistent with findings reported in a review of cancer trials in China (Wu et al., 2020).

Psoriasis is an immune-mediated disease in which the skin inflammatory changes are dependent on immune cells and their cytokines. The prevalence of psoriasis was increasing over time in China, from 0.12% in 1984 to 0.56% in 2019, which is possibly due to the increased awareness of the disease (Cooperative Psoriasis Study Group, 1986; Institute for Health Metrics and Evaluation, 2019; Li, et al., 2021). The TNF-α–IL23–Th17 axis plays a central role in T-cell–mediated plaque psoriasis, while pustular psoriasis is characterized by the increased expression of IL-1β, IL-36α, and IL-36γ transcripts (Rendon, et al., 2019). Multiple cytokines involved in the pathophysiology of psoriasis have already been targeted by approved biologics. Psoriasis accounted for nearly 80% of the identified clinical trials, remaining the most studied dermatological disease treated by biologics. In line with the global trend, targeting cytokines or their cognate receptors that are involved in disease pathogenesis, such as IL-12/23, IL-17A, IL-17F, and IL-17RA, and TNF-α using biologic agents emerged in recent years as a highly effective therapeutic option for patients with moderate-to-severe diseases. Phase III trials of novel drugs in China showed that the 12-week PASI 75 rate was 77.8% for adalimumab (TNF-α mAb) (Cai, et al., 2017), 82.5% for ustekinumab (IL-12/23 mAb) (Zhu, et al., 2013), and 97.7% for secukinumab (IL-17 mAb) (Cai, et al., 2020). The high effectiveness of novel drugs encouraged R&D and led to a high proportion of trials on these targets in China by quite a few local companies. In fact, our study found that all biologics for psoriasis under R&D in the clinical trial stage by these local pharmaceutical companies were biosimilars or “me-too” drugs, when the global psoriasis pipeline has broadened to novel targets, such as ICAM1, NF-kB, REV3 L, ADRA1B, and CCL11 (Bui, et al., 2021). This extremely high proportion of biosimilar R&D on TNF-α and innovative drug R&D on IL-17 by local companies found in our study is typical. Local pharmaceutical companies in China intend to avoid risk, giving priority to follow-on drugs with the potential to be best-in-class (Mullard, 2017). As most of these trials were phase I and initiated from 2018 onwards, the total number of trials is expected to continue to increase in the next few years, and the competition will be more intense. Although this follow-on system can effectively address the huge unmet medical needs of the general population, this may lead to an excess of repetitive clinical trials, thus hampering local pharmaceutical companies in investing in drug innovation.

Our study found that IL-36 (spesolimab) manufactured by a global pharmaceutical company is a new target for psoriasis that is undergoing four clinical trials in China. The biological therapy has been designated as a “breakthrough therapeutic drug” by the CDE (Center for Drug Evaluation, 2021). Three of the four trials registered on the clinicaltrials.gov are multi-country phase II, including China, and one is an open-label phase I trial for safety in China alone. An additional search on clinicaltrials.gov confirmed that all trials on IL-36 (spesolimab) for GPP has involved or been conducted alone in China. China offers a huge patient population that can potentially accelerate clinical trial timelines. Previously, long and inconsistent CTA timelines made it hard to incorporate Chinese study sites into global trials or deterred global companies from operating China-only trials. Limited knowledge of good clinical practices (GCPs) or a limited number of staff with sufficient training and trial experience may also lead to hesitance from global companies including China (Kong, et al., 2017). New “default system” on procedure CTA since 2018 has removed one of the barriers (National Medical Products Administration, 2018). From the “11th Five-Year Plan” to the “13th Five-Year Plan”, with the advancement of innovative drugs and technology, the construction of a new clinical evaluation platform of research and technology and hospital GCP office capacity have also been continuously developed. Based on these improvements, parallel phase I clinical trials in the US and in China have been observed as a new trend in a few therapeutic areas (Xu, 2015), including dermatology, and will contribute to the globalization of the innovated drugs in China.

The pathophysiology of atopic dermatitis is complex and multifactorial, involving elements of barrier dysfunction, alterations in cell-mediated immune responses, IgE mediated hypersensitivity, and environmental factors (David Boothe et al., 2017). Recent genome-wide studies have emphasized Th2 cytokines such as KIF3A, IL-4, and IL-13 as major molecules involved in atopic dermatitis (Tamari and Hirota, 2014; Paternoster, et al., 2011). In China, the prevalence of AD in students aged 6–20 years was only 0.7% in 2000 (Gu, et al., 2000); however, it had reached up to 8.3% (95% CI: 7.6–9.1%) in children aged 3–6 years in Shanghai in 2012 (Xu, et al., 2012). The only available biologic for atopic dermatitis in China is dupilumab targeting at IL-4 and 13, which was approved by the CDE in 2020. A total of six trials for atopic dermatitis were registered over the last 5 years, four of which were registered in 2020, indicating increased awareness of therapeutic gap for atopic dermatitis in China. According to GBD, China had a higher incidence rate of atopic dermatitis than the US as of 2019 (Institute for Health Metrics and Evaluation, 2019). Atopic dermatitis was the second most common dermatological disease in China, which may account for the increasing awareness of atopic dermatitis (Institute for Health Metrics and Evaluation, 2019). Unlike psoriasis, where the PASI 75 rate has achieved greater than 90% with biological treatment (Cai, et al., 2020), the response rate to biologics, in terms of clearing or near-clearing of skin lesions of atopic dermatitis, was only 40% (Beck, et al., 2014). As a result, there are still unmet needs for atopic dermatitis requiring novel treatments with innovative targets. Multiple new agents targeting IL-31, CD134, and Janus kinase proteins have been under investigation for atopic dermatitis (Newsom, et al., 2020). Local companies should explore innovative therapeutic targets in R&D to better meet needs of Chinese atopic dermatitis patients.

In terms of non-cancer dermatological diseases other than psoriasis and atopic dermatitis, very limited trials were performed in China over the past 5 years on biologics, while the global trend was different in these disease areas. Compared with China, where only one trial was registered on the first-generation IgE monoclonal antibody against CSU, other countries have conducted trials on newer generations of IgE monoclonal antibody, such as ligelizumab and UB-221 (Maurer, et al., 2019; Wedi and Traidl., 2021). In addition to IgE, other CSU targets have entered clinical trial stage outside China including IL-5Rα, c-Kit, Siglec-8, and CD200R (ClinicalTrials.gov numbers: NCT04612725, NCT04538794, NCT03436797, and NCT04159701). In addition, novel biologics against certain diseases were only studied outside China by clinical trials such as pyoderma gangrenosum and pemphigus vulgaris (ClinicalTrials.gov numbers: NCT04598477 and NCT03311464). The biologic R&D of these disease areas in China requires further efforts from both global and local pharmaceutical companies.

Our study has the following limitations: 1) Two trials were terminated at a very early stage for variable reasons. We included these trials in our analysis to avoid selection bias, although these trials might not accurately reflect the R&D trend as other trials did. 2) Some biologics had multiple trials registered with the same study phase but different study design. Inclusion of these trials may overestimate the trend of such drug targets or diseases. 3) We only included trials that had at least one study site in China. Since we did not analyze data of trials conducted outside of China or pre-clinical studies, information about the latest drug target and R&D phase may not be complete.

## Conclusion

From our study, we could observe the following trends of dermatological biologic R&D in China: 1) Due to the favorable environment and policy from China regulation agencies, the number of dermatological biologic trials in China surged over the past 5 years, primarily driven by psoriasis trials, with various drug targets. 2) “Me-too” drugs and biosimilars were still the main focus of local pharmaceutical companies. Although it may effectively address the huge unmet medical needs of the Chinese population, it also reflects the lack of innovative R&D capability of local companies. 3) Execution of parallel clinical trials involving China accelerated registration and approval of novel drugs in China and provided more opportunities to the China clinical trial market. 4) Continuous development and maturation of hospital GCP offices provided strong incentive and confidence for pharmaceutical companies to conduct new drug R&D in China.

## Data Availability

Publicly available datasets were analyzed in this study. These data can be found here: https://www.chictr.org.cn; http://www.chinadrugtrials.org.cn/; http://www.clinicaltrials.gov/.
